# Electrochemical behavior, antimicrobial activities, and effect of temperature on micellization of imidazolium monomeric surfactants

**DOI:** 10.55730/1300-0527.3544

**Published:** 2023-01-13

**Authors:** Vinit SHARMA, Tokuma GETAHUN, Manpreet SINGH, Jaswinder KAUR, Nandita THAKUR, Kamal KISHORE

**Affiliations:** 1 School of Advanced Chemical Sciences, Shoolini University, Bajhol, Solan, India; 2 Department of Chemistry, Eternal University, Baru Sahib, Sirmaur, India; 3 Department of Biotechnology, Eternal University, Baru Sahib, Sirmaur, India

**Keywords:** Electrolytes, cationic surfactants, micellization, cyclic voltammetry and antimicrobial activity

## Abstract

In the present study, we herein report the conductance behavior, effect of temperature, and chain-length of two environmentally friendly imidazolium cationic capric and stearic surfactants. The conductance behavior has been carried out in aqueous solvent (H_2_O) at four different temperatures such as 24 °C, 29 °C, 34 °C, and 39 °C. The normal micelles were formed in an aqueous solvent and critical micelle concentration (CMC) can be estimated through conductivity parameters. The expected dependency of the CMC on the alkyl chain length of the 3-(2-(decanoyloxy)ethyl)-1-methyl-1H-imidazol-3-ium-bromide and 3-(2-(octadecanoyloxy)ethyl)-1-methyl-1H-imidazol-3-ium-bromide was demonstrated. It was observed that the graphs of molar conduct activity v/s square root were not linear, which specifies that the synthesized surfactants behave as weak electrolytes in the dilute solutions. The electrochemical characterization of capric and stearic surfactant modified SPCE was studied in 1mM K_3_FeCN_6_ solution. The CS/SPCE and SS/SPCE were shown elevated sensitivity, high stability, and excellent conductivity. Moreover, the antimicrobial behaviors of the synthesized imidazolium cationic surfactants versus various microbial strains were evaluated. Results showed that capric surfactant demonstrated high antibacterial activity against *Escherichia coli* (MIC > 31.5 μg/mL).

## 1. Introduction

Surfactants are materials which decreased the surface parameters of fluid even at a very low concentration [1]. Within specific order to keep away from the contact of the aquo-phobic moieties with water, the single surfactant molecules self-associate to form an array of comprehensive structures, known as micelles [2]. Surfactants are the main compounds used in our regular needs and its application is increasing progressively in every field [3]. The application of surfactants in industry increases day by day at a rate of 3%–4% because of its higher physicochemical possessions, low cost, and affluence of development [4]. Surfactants generally derived from natural fatty acids can be a moral conventional method to synthesize surfactants because of their affluence of manufacturing and good biodegradability [5]. These surfactants are synthesized by using a sustainable and renewable approach with the help of containing biocompatible functional groups which are mainly degraded after use [6]. These surfactants after discarding will release back equivalent carbon [7] to the environment, which is been formerly consumed by plants for making feedstock [8].

Cationic surfactants (hydrophilic moiety is positively charged) acquire many industrialized applications [9]. Some of the most essential application of cationic surfactants includes softeners, cosmetic product, glass cleaners, bleaching aids and dish washing [10] electrode coating and the stabilization of epoxy resin polymer latexes [11] and paper manufacturing [12]. Generally, micellization occurs in front of a threshold surfactant application, known as critical micellar concentration (CMC) [13]. The surfactant particles below CMC are principally distributed as monomers and above they form micelles in surfactant solution [14]. Micellization nature of the cationic surfactants with aquophilic and aquophobic moieties in the molecule has been calculated by use of physical parameters [15]. The CMC is considered as one of the most essential factors used for the comparison of the effectiveness of surface-active agents for their valuable applications [16]. Imidazolium surfactants predominantly are significant molecule employed as corrosion inhibitors and utilized in suspension polymerization for natural resources flotation. These surfactants are mainly synthesized by using of renewable crude substances such as fatty alcohols as well as epichlorohydrin which is an eco-friendly substance [17]. These synthesized surfactants have shown excellent biological activities [18] biocompatibility and biodegradability [19].

Surfactants are widely used in the field of research, mainly in electrochemical studies. Because of their specific amphiphilic structure having anacquophilic head on one side as well asacquophobic tail on the other side, these can be absorbed on the interfaces and on the surfaces [20]. Therefore, surfactants are extensively used in electrochemical detection to get more advanced properties of electrode suspension interface. Adsorption of such molecules on the face of electrodes can effectively change the reaction potential, charge transfer, and diffusion coefficients [21–22].

Therefore, quaternary ammonium-containing surfactants mainly possess antimicrobial activity against bacteria or fungi [23]. These activities of imidazole-based ionic liquids or surfactants are changing with the length of alkyl chains and with varying anions [24]. The antimicrobial activity of surfactants mainly depends upon the chain length of the cationic substituent. Therefore, the antimicrobial activity was found to be completely independent of the anion type [25]. Based on these observations, the present work has been taken with the measurement of the conductance and electrochemical behavior of capric surfactant (CS) and stearic surfactant (SS) in an aqueous solvent. The antimicrobial activity of the prepared imidazolium cationic monomeric surfactants was investigated against bacterial and fungal strains.

## 2. Materials and methods

### 2.1. Chemicals

Stearic acid, capric acid 1-methylimidazole, and 2-bromoethanol were purchased from LobaChemie Pvt. Ltd. Sulphuric acid, chloroform, methanol, ethanol, diethyl ether as well as cold acetone were purchased from CDH Fine Chemicals. Silica gel for TLC and sodium sulfate were purchased from Scientific fishers. All the chemicals were used without any further purification.

### 2.2. Synthesis of capric and stearic imidazolium surfactants

The capric and stearic acid were added to bromo ethanol with a few drops of H_2_SO_4_ and stirred for 2–3 h at 60 °C. The reaction progress was checked by thin layer chromatography (TLC) coated with silica gel layered glass plates. The intermediate esters were instantaneously reacted with 1-methyl imidazole in a 1:1 molar ratio for 30 min at 60 °C and the product was crystallized with diethyl ether and chilled acetone to obtain the product [28].

### 2.3. Conductivity

The calculated number of synthesized surfactants was taken in a standard flask and the solutions were made up by adding the required amount of distilled water. By this method, numerous solutions having different concentrations were prepared to investigate conductance behavior. The conductance measurements of both surfactants in water at four distinct temperatures were carried out with the HANNAH12300 Microprocessor conductivity meter.

### 2.4. Electrochemical measurements

The electrochemical detection was usually performed with FRA-2 μAUTOLAB TYPE III by using a standard three-electrode system: capric surfactant and stearic surfactant modified screen printed carbon electrode (SPCE)as a working electrode; Ag/AgCl as reference electrode as well as counter electrode was also made up of carbon. All the electrochemical measurements by using cyclic voltammetry were performed in 1mM K_3_FeCN_6_ solution.

### 2.5. Antimicrobial activity

The two synthesized imidazolium cationic monomeric surfactants (capric and stearic surfactants) were estimated for their antimicrobial performances by serial dilution method based on nominal inhibitory concentration (MIC) against three bacterial (*Bacillus cereus*, *Escherichia coli*, and *Yersinia enterocolitica)* and one fungal (*Candida albicans*) strains. For this method, twelve test tubes were carried out and only nine were marked as 1–9, and the other three were used as TM (medium), TMC (medium + compound), and TMI (medium + inoculum). All the test tubes were filled with 2 mL of nutrient broth medium by closing with cotton and then purified in an autoclave for 15 lbs/sq. inch pressure. After cooling, 2 mL of the surfactant solution was added to the first test tube via continuous mixing after that 2 mL of this solution was added to the second test tube or from the second to the third. This procedure of serial dilution was sustained up to the ninth test tube after that add 10μL of properly diluted inoculum. To the control test tube TMC, 2 mL of the surfactant sample was added, and 2 mL of this was abandoned for examining the transparency of the medium in the existence of surfactant solution. The growth of the organism in the medium was observed by adding 10 *μ*L inoculum. Consequently, all the test tubes were incubated at 37 °C for 18 h followed via resultant concentrations ranging from 1.9–500 μg/mL. The antibiotics (ampicillin for bacteria as well as miconazole for fungus) were generally used as controls (positive). The shortest concentration of these surfactants to inhibit the growth of fungus and bacteria was generally specified as MIC and expressed as μg/mL [26–27].

## 3. Results and discussion

### 3.1. Preparation and characterization of capric surfactant(CS) and stearic surfactant(SS)

The two monomeric imidazolium cationic surfactants such as 3-(2-(decanoyloxy)ethyl)-1-methyl-1H-imidazol-3-ium-bromide (Capric surfactant) and 3-(2-(octadecanoyloxy)ethyl)-1-methyl-1H-imidazol-3-ium-bromide (Stearic surfactant) have been synthesized from fatty acids as shown in the scheme. Both prepared surfactants have been characterized via FTIR,^1^H-NMR as well as TGA [28].

### 3.2. Conductivity measurements

**Figure 1:**
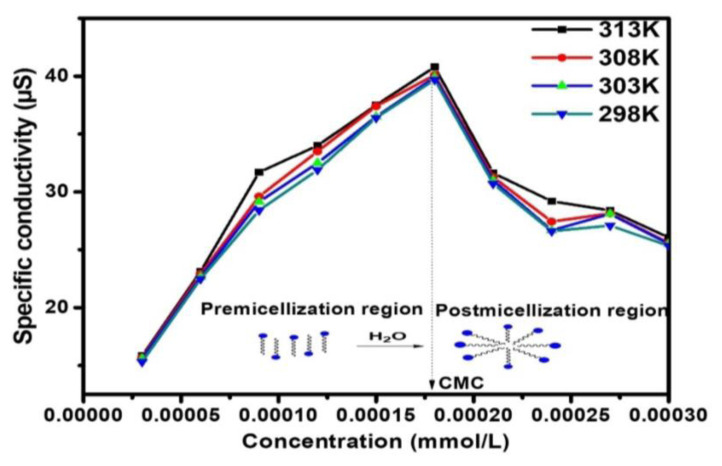
Specific conductivity v/s concentration of stearic surfactant.

The conductometric parameters of capric surfactants (CS)as well as stearic surfactants (SS) were calculated at different temperatures such as 24 °C (298K), 29 °C (303K), 34 °C (308K) as well as 39 °C (313K). The values of specific conductance (μS) of CS in the water rise linearly with increasing concentration and temperatures (Table 1). The graph of specific conductance v/s surfactant concentration of SS has revealed an intersection of dual lines at certainconcentrations relates to critical micellar concentration (CMC) whichdemonstrates that the hydrophobic species starts the formation of ionic micelles (Figure 1).

**Figure 2:**
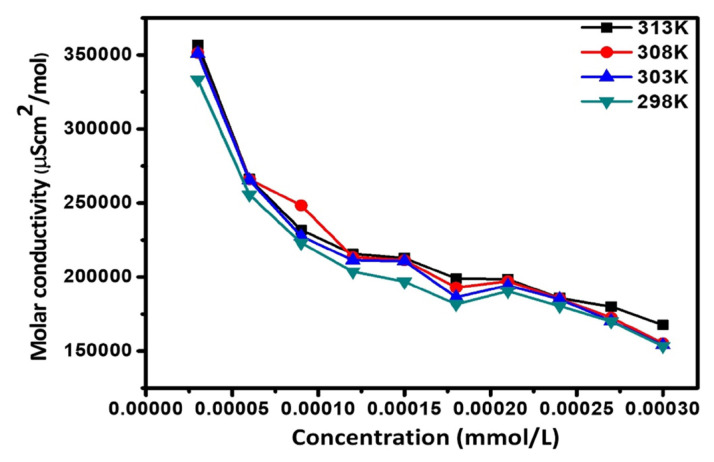
Molar conductivity for capric surfactant.

**Figure 3:**
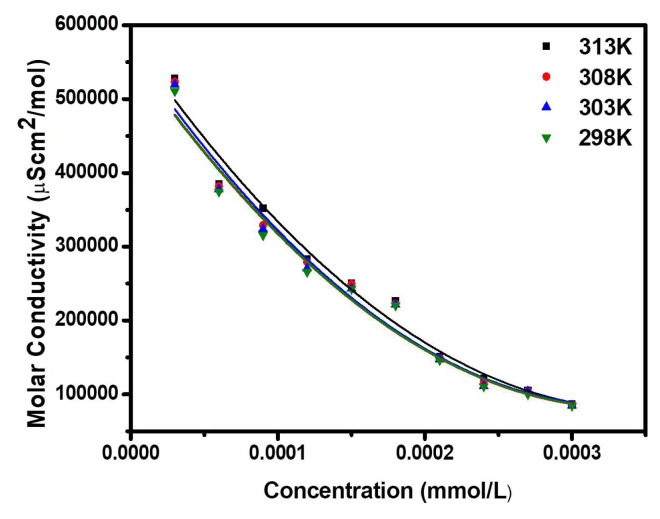
Molar conductivity for stearic surfactant.

The variation of molar conductivity with concentration for both surfactants was not found linear signifying that these surfactants act as a weak electrolyte in H_2_O and so the dissociation constant may be described in terms of Ostwald’s manner [29]. The graphs of molar conductance v/s CS (Figure 2) and SS (Figure 3) concentrations are shown in curved lines. If C demonstrates the concentration and α is the degree of dissociation of synthesized surfactant. Concentrations of the various species could be assumed as:[Fig f9-turkjchem-47-2-375]

**Figure f9-turkjchem-47-2-375:**
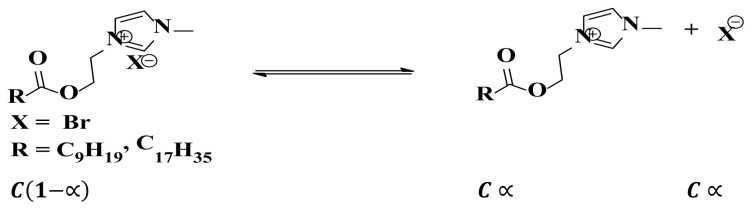


Dissociation constant: K_D_,


(1) $${\text{K}}_{\text{D}}=\frac{\text{C }\propto^{2}}{1-\propto }$$

The α has been calculated efficiently by the conductance percentage, μ/μ_0_ where μ is the molar conductance at a fixed concentration and μ_0_ is the limiting molar conductance at unfixed dilutions. Substituting, α withμ/μ_0_ and after reshuffling equation (1), expressed as:


(2) $$C\mu =K_{D}\mu_{O}^{2}.\frac{1}{\mu }-K_{D}\mu_{O}$$

The values of the μ_o_ of CSas well as SS, and K_D_ have been attained from the plot of slope and intercept between Cμ vs. 1/μ for dilute solutions (Table 2).

The heat of dissociation (ΔH_D_^o^) of CS and SSstated as:


(3) $$\text{log }K_{D}=\frac{-\Delta {\text{H}}_{\text{D}}^{\text{o}}}{2.303\text{RT}+\text{C}}$$

**Figure 4:**
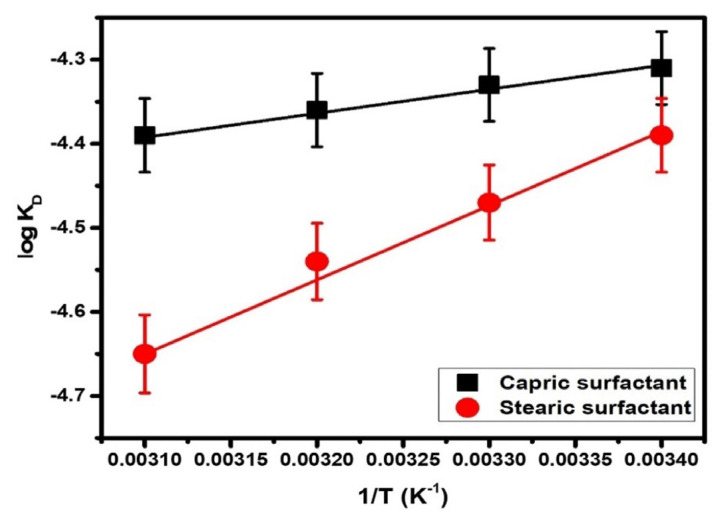
log K_D_ v/s 1/T of capric as well as stearic surfactant.

The values of ΔH_D_^o^ for both surfactants were calculated from the slope of the graphs of log K_D_ v/s 1/T (Figure 4). The dissociation process is exothermic because ΔH_D_^o^ showed negative values (Table 2). The exothermicity of CS and SS increases with an increase in the hydrocarbon chain. The change in free energy of dissociation (ΔG_D_^o^) and standard entropy change (TΔS_D_^o^) per mole for the monomeric surfactant molecules have been calculated by the following equations.


(4) $$\Delta {\text{G}}_{\text{D}}^{\text{o}}=-RT\;\text{log }K_{D}$$

(5) $$\text{T}\Delta {\text{S}}_{\text{D}}^{\text{o}}=\Delta {\text{H}}_{\text{D}}^{\text{o}}-\Delta {\text{G}}_{\text{D}}^{\text{o}}$$

The values of the ΔG_D_^o^ were mainly found to be positive and increasing with an increase in temperature as well as hydrocarbon chain (Table 2). The TΔS_D_^o^ per mole (Table 2) for CS and SS molecules were negative in behavior such as TΔS_D_^o^ < 0. Moreover, TΔS_D_^o^ for dissociation decreases with increasing alkyl chain length and temperature.

In the micellization aggregation process, counter ions principally form micelles after the standard free energy change of micellization (ΔG_M_^o^) for the phase separation model is expressed as:


(6) $$\Delta {\text{G}}_{\text{M}}^{\text{o}}=2RT\;\text{log }X_{CMC}$$

Where X_CMC_ is the CMC of mole fraction,


$$X_{CMC}=\frac{n_{s}}{n_{s}+n_{o}}\approx \frac{n_{s}}{n_{o}}$$

where n_s_is the moles of surfactant and n_o_is number of solution as well as pure solvent. Values of CMC for CS and SS at diverse temperatures are mentioned in Table 2. It is observed that values of CMC were increasing with a rise in temperature and decreased with an increase in the hydrocarbon chain. The micellization process hypothetically occurred when the energy was released. Therefore, the aggregation of the hydrocarbon chain of the surfactant molecules were sufficient to balance the decrease in entropy. The kinetic energy and CMC value of the prepared surfactant were very high at greater temperature. The standard enthalpy changes of micellization (ΔH_M_^o^) for the phase separation model as shown below:


(7) $$\partial \;\text{log }X_{CMC}=\frac{-\Delta {\text{H}}_{\text{M}}^{\text{o}}}{2{\text{RT}}^{2}}$$

**Figure 5:**
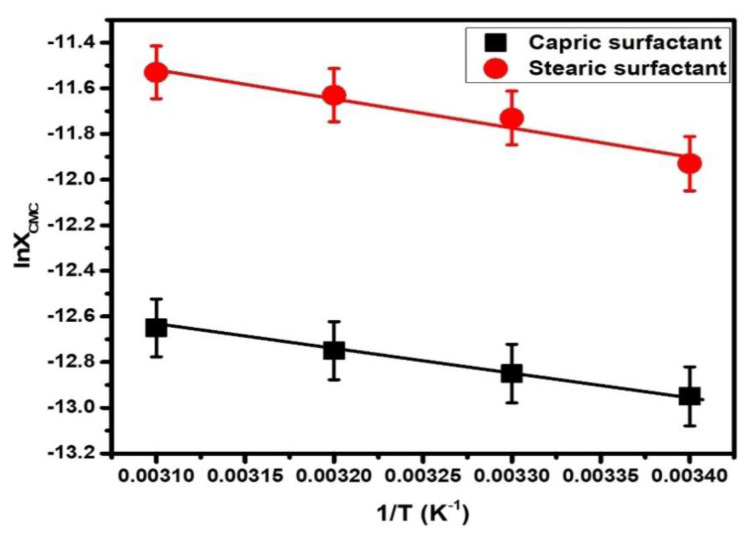
lnX_CMC_ v/s 1/T of capric and stearic surfactant.

The ΔH_M_^o^ is attained from the slope of the plots of lnX_CMC_v/s 1/T (Figure 5). The values of ΔH_M_^o^ of CS and SS are mentioned in (Table 2).

The positive values of ΔH_M_^o^ > 0 specify that the micellization of CS and SS in H_2_O are endothermic. The endothermicity increases with a rise in the chain length of synthesized surfactants. The results specified that the values of TΔS_D_^o^ per mole of surfactant monomers were usually calculated to be positive i.e. TΔS_M_^o^ > 0 (Table 2). The TΔS_M_^o^ increases with an increase in temperature and decreases with hydrocarbon chain length. The ΔG_M_^o^ (Table 2) for CS and SS, is negative (ΔG_M_^o^ < 0) which increases with a rise in temperature. All results were found to be comparable with other literature [30–31].

### 3. 3. Micelle solutions models of the electrode

**Figure 6:**
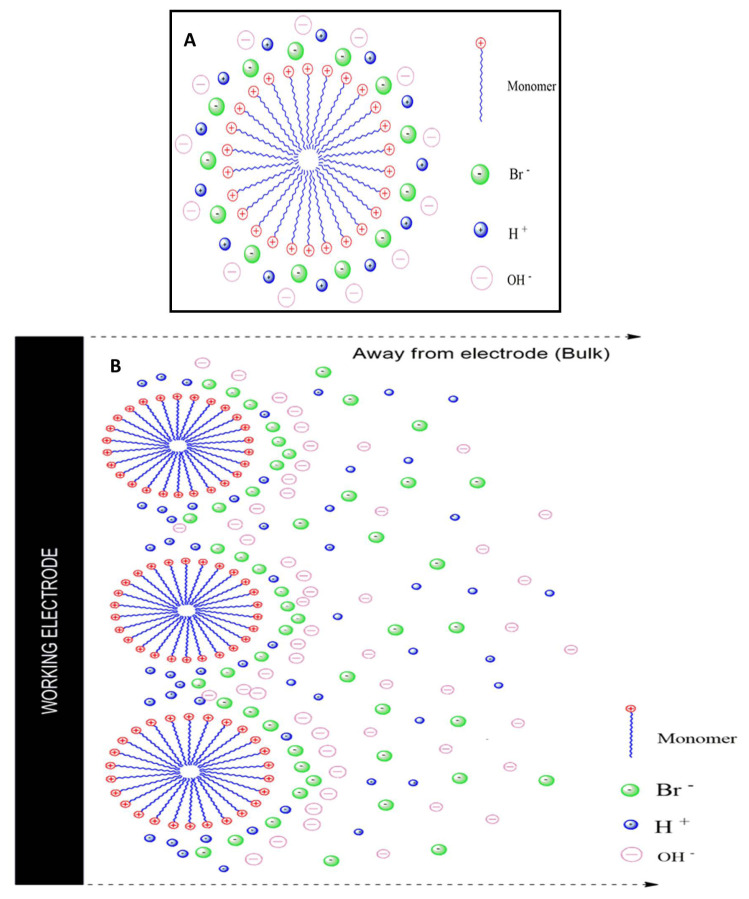
(A) Spherical micelle models, (B) Plausible mechanism for micelle solutions at the working electrode.

The spherical micelles were formed when the concentration of surfactant is above the critical micellar concentration (CMC). The development of micelles produced the interface within the aquaphobic area holding the surfactant ends in the aqueous standard Figure 6 (A). Surfactant solutions show electrostatic repulsion through OH^−^ by using the negative charges of the cationic micellar surface. Moreover, the H^+^ ion and the negative charges of cationic micelles appeal to each other. Therefore, micellar anions adsorbed nearby the working screen-printed carbon electrode (SPCE) and create some space for OH^−^. Consequently, only the OH^−^ ions can be oxidated, which may lead to an increase in the anodic peak current value of the prepared surfactant. Based on the cyclic voltammograms (CV), we mentioned the plausible mechanism for the behavior of surfactant micelle solutions nearby the SPCE electrode as shown in Figure 6 (B).

### 3. 4. Electrochemical behaviors of bare/SPCE, CS/SPCE and SS/SPCE

**Figure 7:**
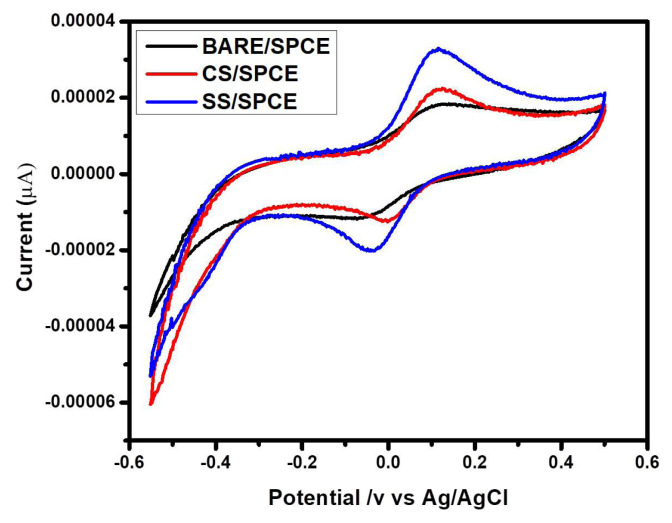
Cyclic voltammograms of 1 mM K_3_FeCN_6_ at bare/SPCE and CS and SS modified SPCE at a scan rate of 50 mVs^−1^.

The electrochemical activities of bare, capric surfactant (CS), and stearic surfactant (SS) modified SPCE in 1mM potassium ferrocyanide (K_3_FeCN_6_) at a 50 mVs^−1^ scan rate (Figure 7). The 0.00018 mmol/L concentration of CS and SS solutions was used for the electrode modification of screen-printed carbon electrode (SPCE). Consequently, a few drops of nafion (binder) were added to the solution followed by 1 h of sonication. The cyclic voltammograms were generally recorded by dropping 60 μL of testing solution(K_3_FeCN_6_) on the surface of the bare, CS, and SS modified electrode through a micropipette. The CV graphs were recorded in the potential range of −0.6 to 0.6 V. The peak current of SPCE was increased when modified with CS (red), SS (blue) as compared to that of bare/SPCE (black). The peak potential for two modified electrode systems were such as anodic (Epa) = 0.118V and cathodic (Epc) = 0.039V peak potentials for SS/SPCE, while Epa = 0.0115V as well as Epc = 0.004V for CS/SPCE. Therefore, these redox peak potentials indicated that the SS/SPCE showed a high value of anodic peak current as compared to that of CS/SPCE and bare/SPCE due to the presence of higher chain length. The surface modified SPCE electrodes with surfactants increase with increasing carbon chain length [32]. Therefore, both prepared surfactants exhibited good electrochemical performances with higher conductivity.

### 3.6. Antimicrobial activity

Nowadays, cationic surfactants are known to act as effective biocides properties and antibacterial agents [33]. The antimicrobial activities of molecules can be evaluated by different methods [34]. In this study, the effects of the imidazolium cationic surfactants (capric and stearic surfactants) on the selected microbial strains were investigated. The MIC values of these surfactants against *B. cereus*, *E. coli*, *Y. enterocolitica*, *C. albicans* are reported in Table 3. The results demonstrated that the prepared imidazolium cationic surfactants showed antimicrobial activities against the tested microbial strains. Interestingly, significant antibacterial activity was observed for capric surfactant, with a MIC value of >31.5 μg/mL against the Gram-negative bacterium, *E. coli*. However, the MIC value of both synthesized surfactants against *B. cereus*, *Y. enterocolitica*, and *C. albicans* as well as stearic surfactant against *E. coli* was >62.5 μg/mL (Table 3).

The fatty acids from which these surfactants were synthesized have been known for their antimicrobial activities which were dependent on their alkyl chains [35]. The antimicrobial activity results against *E. coli* also showed that the biological activities of the synthesized surfactants depend on the alkyl chain length. The antibacterial activity of the two surfactants increased by decreasing the alkyl chain length. This may be observed that the large decrease in the lipophilicity of the molecules showed the existence of a hydrophobic chain, which helped to take less time for crossing the cell membrane and hence activity increases. Moreover, the antimicrobial activity is depending on the cationic moiety of the cationic surfactants as well. The microbial cell wall has a net negative charge due to the presence of teicholic acid attached to either peptidoglycan or to the underlying plasma membrane, or the outer covering of the cell wall contains phospholipids and lipopolysaccharides which provide a negative charge to the outer surface. The cationic moieties of the surfactants are attracted by the negative charge of the microbial cell wall; this causes strong adsorption of the surfactants due to ionic interactions with phospholipids of the microbial cell wall. Ultimately, the protective barrier (cell wall) loses its role and active ingredients are easily able to enter the cytoplasmic membrane which is responsible for the antimicrobial activities of these cationic surfactants [36–37]. In contrast, CS and SS showed comparable reasonable activity against *B. cereus* (Gram-positive bacterium), *Y. enterocolitica* (Gram-negative bacterium), and *C. albicans* (fungus).

## 4. Conclusion

It may be concluded, that the micellization process of CS and SS is reliable with ΔH_M_^o^ > 0, ΔG_M_^o^ < 0, and TΔS_M_^o^ > 0, whereas the dissociation process of these molecules’ agreements with ΔH_D_^o^ < 0, ΔG_D_^o^ > 0, and TΔS_D_^o^ < 0. The critical explanations of thermodynamic results specify that the micellization process is spontaneous and favored over the dissociation process. The results showed that the thermodynamics of dissociation and micellization can be explained suitably by using the phase separation model. The CS/SPCE and SS/SPCE displayed tremendous electrochemical performance as compared to that of bare/SPCE and showed superior stability, excellent conductivity, and good sensitivity. Additionally, both imidazolium cationic surfactants gave good antimicrobial activity. However, capric surfactant showed strong antibacterial activity than stearic surfactant towards Gram-negative *E. coli* bacterium.

## Figures and Tables

**Scheme:**
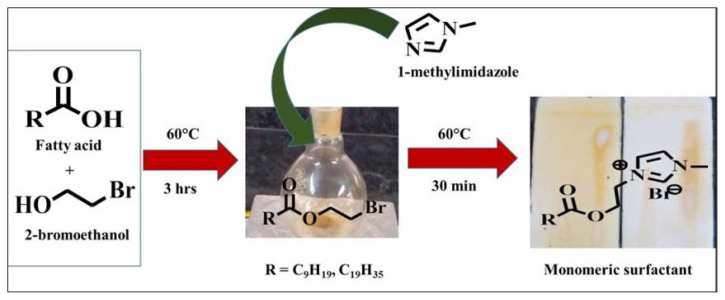
Synthesis of CS and SS imidazolium surfactants.

**Table 1:** Specific conductivity vs molar conductance.

Concen-tration (mmol/L)	Capric surfactant							
	298K		303K		308K		313K	
	Specific conductance (μS)	Molar conductance (μScm^2^/mol)	Specific conductance (μS)	Molar conductance (μScm^2^/mol)	Specific conductance (μS)	Molar conductance (μScm^2^/mol)	Specific conductance (μS)	Molar conductance (μScm^2^/mol)
0.00003	10.00	333333.3	10.53	351000.0	10.54	351333.3	10.71	357000.0
0.00006	15.34	255666.6	15.93	265500.0	15.96	266000.2	15.98	266333.3
0.00009	20.08	223111.1	20.48	227555.5	20.56	248444.4	20.86	231777.7
0.00012	24.44	203666.6	25.37	211416.6	25.62	213500.0	25.88	215666.6
0.00015	29.53	196866.6	31.61	210733.3	31.70	211333.3	31.92	212800.0
0.00018	32.71	181722.2	33.56	186444.4	34.72	192888.8	35.82	199000.0
0.00021	40.01	190532.8	40.81	194333.3	41.41	197190.4	41.7	198571.4
0.00024	43.31	180458.3	44.43	185125.0	44.52	185500.0	44.61	185875.0
0.00027	45.90	170000.0	46.00	170370.3	46.61	172629.6	48.62	180074.7
0.00030	46.01	153366.6	46.22	154066.6	46.61	155366.6	50.31	167700.0
**Stearic surfactant**								
0.00003	14.32	510666.7	14.58	486000.0	15.69	523000.0	15.83	527666.6
0.00006	22.48	374666.7	22.65	377500.0	22.90	381666.7	23.11	385166.7
0.00009	28.41	315666.7	29.15	323888.9	29.60	328888.9	31.70	352222.2
0.00012	31.90	265833.3	32.50	270833.3	33.50	279166.7	34.00	283333.3
0.00015	36.40	242666.7	36.50	243333.3	37.40	249333.3	37.50	250000.0
0.00018	39.70	220555.6	39.90	221666.7	40.10	222777.8	40.80	226666.7
0.00021	30.72	146285.7	31.00	147619	31.28	148952.4	31.63	150619.0
0.00024	26.61	110875	26.68	111166.7	27.43	114291.7	29.19	121625.0
0.00027	27.09	100333.3	28.1	104074.1	28.14	104222.2	28.40	105185.2
0.00030	25.34	84466.6	25.51	85033.3	25.61	85366.6	26.05	86833.3

**Table 2:** Thermodynamic parameters of capric as well as stearic surfactant and the heat of dissociation (ΔH_D_^o^) calculated from the slope of the plots of log K_D_ v/s 1/T and standard enthalpy change of micellization (ΔH_M_^o^) found from slope of the plots of lnX_CMC_ v/s 1/T.

Physical parameters	CS				SS			
	298K	303K	308K	313K	298K	303K	308K	313K
μ**_o_x10^3^** (μScm**^2^/mol)**	450	475	482	500	650	670	690	700
**CMC x 10** **^−4^** **(mol/L)**	2.346	2.531	2.897	3.265	1.752	1.795	1.818	1.865
**K** **_D_** ** x10** **^−5^**	4.90	4.70	4.40	4.10	4.10	3.40	2.90	2.30
ΔG**_D_^o^ (kJ/mol)**	24.59	25.11	25.69	26.29	25.03	25.92	26.76	27.79
**−**ΔG**_m_^o^ (kJ/mol)**	64.23	64.92	65.31	65.74	64.23	64.92	64.99	65.74
**−TΔS** **_D_** **^o^** ** (kJ/mol)**	31.61	32.13	32.71	33.31	45.45	46.34	47.18	48.21
**−TΔS** **_M_** **^o^** ** (kJ/mol)**	41.37	42.06	42.45	42.88	38.37	39.06	39.13	39.88
**−**ΔH**_D_^o^ (kJ/mol)**	7.02				20.42			
**ΔH**_M_^o^ **(kJ/mol)**	22.86				25.86			

**Table 3:** MIC values of CS and SS against microbial strains.

Compounds	MIC (μg/mL)			
	*Bacillus cereus*	*Escherichia coli*	*Yersinia enterocolitica*	*Candida albicans*
**Capric Surfactant**	>62.5	>31.5	>62.5	>62.5
**Stearic Surfactant**	>62.5	>62.5	>62.5	>62.5
**Ampicillin**	39	78.1	39	-
**Miconazole**	-	-	-	78.1

## References

[1] Demirbas MF Balat M Balat H Potential contribution of biomass to the sustainable energy development Energy Conversion and Management 2009 50 7 1746 60

[2] Puvvada S Blankschtein D Molecular and thermodynamic approach to predict micellization, phase behavior and phase separation of micellar solutions. I. Application to nonionic surfactants The Journal of Chemical Physics 1990 92 6 3710 24

[3] Altenbaher B Šostar Turk S Fijan S Biodegradation of typical laundry wastewater surfactants–a review Tekstil: časopiszatekstilnu i odjevnutehnologiju 2014 63 3–4 107 12

[4] Foley P Beach ES Zimmerman JB Derivation and synthesis of renewable surfactants Chemical Society Reviews 2012 41 4 1499 518 22006024 10.1039/c1cs15217c

[5] Mudge SM DeLeo PC Estimating fatty alcohol contributions to the environment from laundry and personal care products using a market forensics approach Environmental Science: Processes & Impacts 2014 16 1 74 80 24220601 10.1039/c3em00418j

[6] Gameiro MD Goddard A Taresco V Howdle SM Enzymatic one-pot synthesis of renewable and biodegradable surfactants in supercritical carbon dioxide (scCO_2_) Green Chemistry 2020 22 4 1308 18

[7] Sharma V Getahun T Verma M Villa A Gupta N Carbon based catalysts for the hydrodeoxygenation of lignin and related molecules: A powerful tool for the generation of non-petroleum chemical products including hydrocarbons Renewable and Sustainable Energy Reviews 2020 133 110280

[8] Bhadani A Iwabata K Sakai K Koura S Sakai H Sustainable oleic and stearic acid based biodegradable surfactants RSC advances 2017 7 17 10433 42

[9] Wei D Zhang H Cai L Guo J Wang Y Calcined mussel shell powder (CMSP) via modification with surfactants: Application for antistatic oil-removal Materials 2018 11 8 1410 30103511 10.3390/ma11081410 PMC6119888

[10] Tyagi S Tyagi VK Novel cationic Gemini surfactants and methods for determination of their antimicrobial activity–review Tenside Surfactants Detergents 2014 51 5 379 86

[11] Tsubone K Ogawa T Mimura K Surface and aqueous properties of anionic gemini surfactants having dialkyl amide, carboxyl, and carboxylate groups Journal of Surfactants and Detergents 2003 6 1 39

[12] Geng T Zhang C Jiang Y Ju H Wang Y Synergistic effect of binary mixtures contained newly cationic surfactant: Interaction, aggregation behaviors and application properties Journal of Molecular Liquids 2017 232 36 44

[13] De Vendittis E Palumbo G Parlato G Bocchini V A fluorimetric method for the estimation of the critical micelle concentration of surfactants Analytical Biochemistry 1981 115 2 278 86 7304960 10.1016/0003-2697(81)90006-3

[14] Vulliezlenormand B Eiselé JL Determination of detergent critical micellar concentration by solubilization of a colored dye Analytical Biochemistry 1993 208 2 241 3 8452216 10.1006/abio.1993.1039

[15] Palmer M Hatley H The role of surfactants in wastewater treatment: Impact, removal and future techniques: A Critical Review Water research 2018 147 60 72 30300782 10.1016/j.watres.2018.09.039

[16] Rather MA Rather GM Pandit SA Bhat SA Bhat MA Determination of cmc of imidazolium based surface active ionic liquids through probe-less UV–vis spectrophotometry Talanta 2015 131 55 8 25281072 10.1016/j.talanta.2014.07.046

[17] Patial P Shaheen A Ahmad I Synthesis of ester based cationic pyridiniumgemini surfactants and appraisal of their surface active properties Journal of Surfactants and Detergents 2013 16 1 49 56

[18] Zakharova LY Pashirova TN Doktorovova S Fernandes AR Sanchez-Lopez E Cationic surfactants: Self-assembly, structure-activity correlation and their biological applications International Journal of Molecular Sciences 2019 20 22 5534 31698783 10.3390/ijms20225534 PMC6888607

[19] Lourith N Kanlayavattanakul M Natural surfactants used in cosmetics: glycolipids International Journal of Cosmetic Science 2009 31 4 255 61 19496839 10.1111/j.1468-2494.2009.00493.x

[20] Atta NF Galal A Ahmed RA Poly (3, 4-ethylene-dioxythiophene) electrode for the selective determination of dopamine in presence of sodium dodecyl sulfate Bioelectrochemistry 2011 80 2 132 41 20709605 10.1016/j.bioelechem.2010.07.002

[21] Li Y Hao J Li G Electrochemical behavior of cationic-anionic surfactant solutions by cyclic voltammetry Journal of Dispersion Science and Technology 2006 27 6 781 7

[22] Sharma V Kaur GA Gupta N Shandilya M Growth mechanism of rGO/CDs by electrospun calcination process: Structure and application FlatChem 2020 24 100195

[23] Garcia MT Ribosa I Perez L Manresa A Comelles F Aggregation behavior and antimicrobial activity of ester-functionalized imidazolium-and pyridinium-based ionic liquids in aqueous solution Langmuir 2013 29 8 2536 45 23360222 10.1021/la304752e

[24] Pernak J Sobaszkiewicz K Mirska I Anti-microbial activities of ionic liquids Green Chemistry 2003 5 1 52 6

[25] Kanjilal S Sunitha S Reddy PS Kumar KP Murty US Synthesis and evaluation of micellar properties and antimicrobial activities of imidazole-based surfactants European Journal of Lipid Science and Technology 2009 111 9 941 8

[26] Zaki MF Tawfik SM Synthesis, surface properties and antimicrobial activity of some germanium nonionic surfactants Journal of Oleo Science 2014 ess14052 10.5650/jos.ess14052 25132086

[27] Negm NA Tawfik SM Characterization, surface properties and biological activity of some synthesized anionic surfactants Journal of Industrial and Engineering Chemistry 2014 20 6 4463 72

[28] Sharma V Bhatia C Singh M Singh C Upadhyaya SK Kishore K Synthesis, thermal stability and surface activity of imidazolium monomeric surfactants Journal of Molecular Liquids 2020 308 113006

[29] Kishore K Upadhyaya SK Gupta A Thakur N Viscosity measurements of Terbium Octanoate in mixed organic solvent *Asian Journal of Advanced Basic Sciences* 2013 1 1 51 7

[30] Kishore K Upadhyaya SK Investigation Into the Conductance, Micellization and Dissociation Behaviour of Terbium Caprylate and Caprate in 60/40 Benzene-methanol Mixture (v/v) PortugaliaeElectrochimicaActa 2010 28 4 213 9

[31] Wani FA Khan AB Alshehri AA Malik MA Ahmad R Synthesis, characterization and mixed micellization study of benzene sulphonate based gemini surfactant with sodium dodecyl sulphate Journal of Molecular Liquids 2019 285 270 8

[32] Dai Z Ju H Effect of chain length on the surface properties of ω-carboxy alkanethiol self-assembled monolayers Physical Chemistry Chemical Physics 2001 3 17 3769 73

[33] Tantawy AH Abo-Riya MA Abdallah SM El-Dougdoug W Novel cationic surfactants based on waste frying oil for cleaning water surface from petroleum films: Synthesis, antimicrobial and surface properties Journal of Molecular Liquids 2018 253 36 44

[34] Getahun T Sharma V Gupta N The genus *Laggera* (Asteraceae)–ethnobotanical and ethnopharmacological information, chemical composition as well as biological activities of its essential oils and extracts: a review Chemistry & Biodiversity 2019 16 8 1900131 10.1002/cbdv.201900131 31173470

[35] Getahun T Sharma V Gupta N Chemical composition, antibacterial and antioxidant activities of oils obtained by different extraction methods from *Lepidiumsativum* L. seeds Industrial Crops and Products 2020 156 112876

[36] Denyer SP Mechanisms of action of antibacterial biocides International Biodeterioration & Biodegradation 1995 36 3–4 227 45

[37] Wölfel L Mach F Chattopadhyay SP Comparative cytologic studies on the effect of cetyltrimethylammonium bromide on bacterial cells Zentralblatt fur Mikrobiologie 1985 1 140 8 631 9 3938143

